# Contamination, Sources, and Health Risks Associated with Soil PAHs in Rebuilt Land from a Coking Plant, Beijing, China

**DOI:** 10.3390/ijerph16040670

**Published:** 2019-02-25

**Authors:** Wei Cao, Liqin Yin, Dan Zhang, Yingying Wang, Jing Yuan, Yi Zhu, Junfeng Dou

**Affiliations:** 1 College of Water Sciences, Beijing Normal University, Beijing 100875, China; caowei@mail.bnu.edu.cn (W.C.); 18435154253@163.com (L.Y.); wangyingyingfei@126.com (Y.W.); xyvy-8945@163.com (J.Y.); 2 Beijing Municipal Research Institute of Environmental Protection, National Engineering Research Center of Urban Environmental Pollution Control, Beijing Key Laboratory for risk modeling and remediation of contaminated sites, Beijing 100037, China; zhangdan615@126.com

**Keywords:** Coking plant, PAHs, residential land, incremental lifetime cancer risks, health risks assessment

## Abstract

This study investigated the polycyclic aromatic hydrocarbon (PAH) pollution in the reconstructed land of an abandoned industrial site: a coking plant in Beijing. To meet the needs of urban development, many factories have had to be relocated from city centers, and abandoned industrial sites often need to be transformed into residential land or urban green space through a series of restoration measures. It is necessary to study the level of residual pollutants and potential risks associated with industrial reconstructed land. The concentration of 16 PAHs in the study area ranged from 314.7 to 1618.3 µg/kg, and the average concentration was still at a medium pollution level; the concentration of PAHs in the original coking workshop had the highest levels (1350.5 µg/kg). The PAHs in the soil were mainly low-ring aromatics, especially naphthalene and phenanthrene. The isomer method and principal component analysis indicated that PAHs in the topsoil were the result of coal and biomass combustion. The seven carcinogenic PAHs were the main contributors to the total toxicity equivalence. The genetic toxicity of benzo[a]pyrene was relatively low, and the results were related to the concentration level. There were potential carcinogenic risks for people of varying ages in this residential area. In total, gender differences were small, and the comprehensive lifetime cancer risk level was still acceptable. For the remaining plots at the study site, the daily intake of PAHs by construction workers was between 0.74–2.31 ng/kg bw/day, which requires further evaluation about ignored area occupational exposure to environmental pollutants.

## 1. Introduction

Polycyclic aromatic hydrocarbons (PAHs) in soil, water, and atmosphere have aroused extensive concern owing to their carcinogenic, teratogenic, and mutagenic factors, as well as environmental pollution [1,2,3,4,5]. In general, PAHs in urban environments originate from human activities and technology, such as automobile exhaust, power generation from coal and fossil fuels, industrial processing, and chemical manufacturing [6,7,8,9]. Due to their lipophilicity and hydrophobicity, PAHs are easily adsorbed onto soil particles, which reduces the likelihood of degradation [10]. PAHs have become a major category of urban pollutants, with potential carcinogenic risks to residents.

As the capital, Beijing is the political, cultural, and commercial center of China. The ancient Forbidden City expanded outward from six concentric circles, each of which was separated by a circular highway. Along with rapid urbanization, the population of Beijing now exceeds 21 million. Due to recent and severe air pollution in urban areas, as well as preparations for the Beijing Winter Olympics in 2022, Beijing has launched a series of measures to improve urban environmental quality. Initiatives include restricting vehicle use based on tail number, encouraging the use of new energy vehicles, using clean energy for central heating, and moving industrial factories out of the city. For example, the Beijing Coking Plant was built in 1959 and located in Fatou area, outside the East Fourth Ring of Chaoyang District. This facility was once a major production base for pipeline gas and the largest coke supply and export base in Beijing and China. It was closed in 2006 because of the need for urban development and the Beijing Olympic Games, and its workshop and production equipment were moved to Tangshan in Hebei province. Subsequently, the city reserved the coking plant land, leaving a large area of brownfield land [11]. As a result of coal chemical and coking, the main pollutants left in the soil were PAHs, benzene series, and petroleum hydrocarbons. Through a series of soil remediation measures, some areas were converted into residential land in 2013, but the land may still pose a carcinogenic risk for human health.

Numerous studies have assessed the pollution and health risks of soil PAHs in urban and industrial areas. By studying the distribution and potential sources of PAHs in the soil near the three coal-burning areas in South Africa, Okedeyi et al. [12] found that the PAH pollution level in the surrounding soil was high, with a strong carcinogenic risk. Zhang et al. [13] analyzed PAHs in the atmospheric dust of the industrial corridor in Hubei province, China, and discovered that the PAHs were mainly derived from motor vehicles and biomass and coal combustion, which can cause lifetime carcinogenic risk to residents. Suman et al. [14] observed that the PAHs emitted by urban traffic are derived mainly from fossil fuel combustion vehicles, and the carcinogenic effect of PAH load in traffic soil was nearly 6.15 times higher than that in control/rural soil. Jia et al. [15] found that the PAH concentration of industrial area soil in suburban Shanghai was positively correlated with organic matter content, and the PAHs in road dust may have been an important source. However, there has been little research concerning the potential health risks associated with urban residential land in industrial areas. The purpose of this study was to evaluate PAH pollution and its source, as well as the health risk and potential lifetime carcinogenic risk of residual contaminants on local residents and construction workers’ occupational exposure during the construction period, and provide valuable information for the benefit of human health and urban development.

## 2. Materials and Methods

### 2.1. Chemicals

There are 16 priority controlled PAHs designated by the United States Environmental Protection Agency (USEPA), which include naphthalene (Nap), acenaphthylene (Acy), acenaphthene (Ace), fluorene (Fl), phenanthrene (Phe), anthracene (Ant), fluoranthene (Flu), pyrene (Pyr), benz[a]anthracene (BaA), chrysene (Chr), benzo[b]fluoranthene (BbF), benzo[k]fluoranthene (BkF), benzo[a]pyrene (BaP), indeno[1,2,3-cd]pyrene (InP), dibenz[a,h]anthracene (DBA) and benzo[g,h,i]perylene (BP), which were all purchased from American AccuStandard, Inc. All of the used solvents were chromatographic grade and purchased from Fisher Scientific, USA. The pretreatment for silica gel and glassware has been reported in a previous study [16].

### 2.2. Soil Sampling

Our research focused on an abandoned coking plant located in the Fatou area of Chaoyang District, Beijing. Some areas of the coking plant had been converted into residential land after the completion of soil remediation in 2013. The Beijing Public Rental Housing Reconstruction Project (PRHRP) was planned for other areas of the coking plant construction, which may begin between 2018–2022. It is expected that all parts of the plant would be converted into residential land.

Since the source of pollutants might be closely related to the location of the coking plant production workshops, we divided the coking plant into eight regions according to the location of the production workshops. The construction site for the new project (PRHRP) is located in the original workshops 1–4 of the coking plant (CP), while workshops 5–8 are on residential land (RL). The previous plant include a storage workshop (SW), gas workshop (GW), coking workshop (CW), depuration workshop (DW), repair workshop (RW), power workshop (PW), water treatment workshop (WW), and tar workshop (TW).

**Figure 1:**
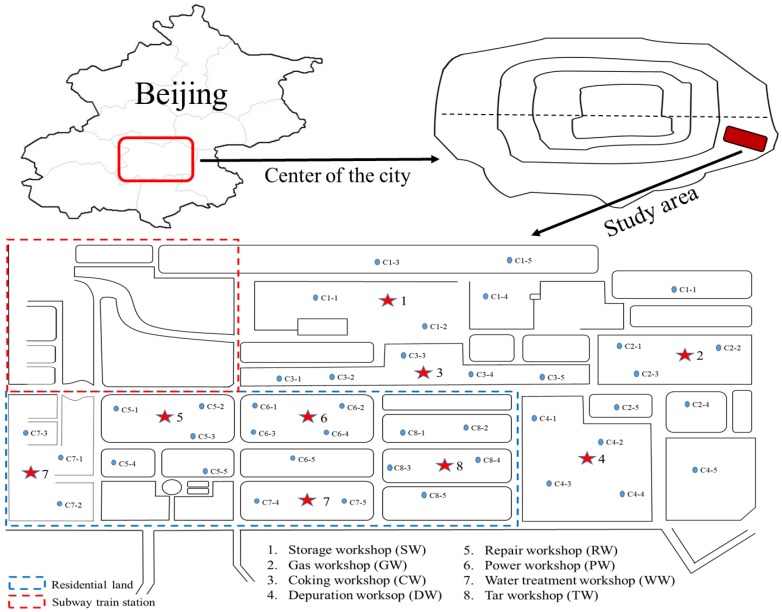
Schematic of sampling sites in study area, Beijing, China.

In April 2018, five soil samples (0–20 cm depth) were collected randomly within a 50-m radius of the different workshops in the coking plant, for a total of 40 sampling points. There had been no precipitation for the week prior to sampling. The specific sampling point positions are shown in Figure 1. After sampling, the soil samples were transported to the laboratory, naturally air-dried, passed through a 100-mesh sieve to remove non-soil materials such as plant roots and stones, and stored at −4 °C.

### 2.3. PAH Analysis

To determine PAH in soils, four grams of soil was extracted using 15 mL of acetone and n-hexane solvent (1:1) in a special extraction cartridge. Microwave extraction was completed in the following manner. The temperature was raised to 100 °C at 10 °C/min, and the sample was extracted at 100 °C for 15 minutes using 1600 W of power. After the sample had cooled to room temperature, the extracting solvent was transferred to the inverted specimen jar. The extraction cartridge was rinsed three times with n-hexane; then, the washing liquid was transferred to the inverted specimen jar to reduce the loss of the target. After the microwave extraction, the sample was purified in the chromatographic column, from top to bottom, using 10 cm of activated silica gel, two cm of anhydrous sodium sulfate, and bottom-stacked absorbent cotton. Then, the extracting solvent was applied to the chromatographic column and eluted with 60 mL of dichloromethane and n-hexane (1:1). The sample was concentrated to 0.3 mL via a rotary evaporator and a pressure blowing concentrator. Targets’ volumes were increased to 0.5 mL using n-hexane, and then frozen prior to the final analysis.

The PAH eluate was analyzed using an Agilent 6890 gas chromatograph (GC) equipped with an HP-5 capillary column (30-m length, 0.25-mm inner diameter, 0.25-µm film thickness; J & W Scientific Inc., Folsom, CA, USA) and a 5975C mass selective detector (MS). Helium was used as a carrier gas and was injected at a rate of one mL/min without splitting. For the program setting, the temperature was programmed to 60 °C, held for one minute, and then raised to 110 °C at 20 °C/min. Afterwards, the temperature was raised to 290 ℃ at 4 ℃/min and held for 20 minutes; the detector temperature was 290 °C. A mixed standard (800 μg/L) was first analyzed in full-scan mode (m/z 50–500) for the identification of the quantification ion, confirmation ion, and retention time of each compound. For the selected ion monitoring (SIM) mode, the chromatogram was divided into several retention time windows on the basis of the results with the full-scan mode. For each compound, quantification and confirmation ions were used for qualitative analysis, and quantification ions were used for quantitative analysis. Detailed information of the individual PAH, including selective ions, linear equations, and peak time, are shown in Table S1.

### 2.4. Quality Assurance and Quality Control

In terms of the Quality Assurance and Quality Control (QA/QC) system, the entire analysis process was monitored using the method blank, matrix spikes, and parallel sampling. Blank and matrix spiked samples’ material used the soils from the school’s flower house of Beijing Normal University. In addition, the pretreatment of the sample and the effects of the matrix were tested with the recovery rate indicator. The detection limit of the analytical method was set between 0.032–0.76 µg/kg. Only trace targets were detected in the method blank, and the reagents that were used were all chromatographic grade. The average recovery rate of the sample, based on the added matrix, was 78–115%; Phe had the best recovery rate, and the recovery rates of Nap and BaA were relatively low, but overall were in a reasonable range. The standard deviations were between 3.5–16.7%. A total of 0.5 µg/kg of each of the d-PAHs was spiked in each four-g soil sample (including the matrix-spiked sample) before the extraction. Both blank and matrix spikes had also undergone the entire pretreatment process. The recoveries for d-PAHs were acenaphthene-d10, 83.2 ± 17.5%; phenanthrene-d10, 76.7 ± 9.4%; and perylene-d12, 91.8 ± 6.9%.

### 2.5. Genotoxicity Analysis

As an auxiliary chemical analysis technique for environmental risk assessment [17], the application of whole-cell bioreporters has unique advantages, especially in terms of reflecting the bioavailability and toxicity of pollutants [18]. They can also be used for in situ or online measurements by direct contact with environmental media [19]. Sun et al [20] evaluated the Nap in the PAH-contaminated site via both an ADPWH_Nah bioreporter and GC/MS analysis, and demonstrated the reliability and feasibility of the bioreporter in environmental monitoring and bioavailability assessment. Therefore, in this study, we conducted a genotoxicity test from the perspective of biological effects.

The ADP1_recA is a bioreporter that fuses the promoter gene recA with the reporter gene luxCDABE on the chromosome of Acinetobacter baylyi to express the bioluminescence in the presence of genotoxins and determine the level of contamination [21]. It was used as a bacterial test strain, while mitomycin C (MMC) and BaP were used as positive controls. We prepared a series of standard soil samples using concentrations of MMC and BaP. First, 200 mg of each soil sample was dissolved in five mL of ultrapure water and sonicated at 40 kHz for 300 s. Then, 198 µL of the bioreporter suspension, under suitable conditions (suitable growth conditions and the final concentration of ADP1_recA before the mixing with supernatant of soil solution), and two µL of the supernatant of the soil solution were mixed evenly in a 96-well microplate (black/clear bottom) for measuring bioluminescence and OD600. Each soil sample was performed in triplicate. Using the background soil of the sampling region as a negative control, the same luminescence test was performed on the actual contaminated soil samples. The luminescence intensity for the unit cells was calculated, and the induction ratio was evaluated by dividing the relative luminescence by the control group.

### 2.6. Health Risk Assessment

Toxic equivalency factors (TEFs) have been widely used to calculate toxic equivalent quantities (TEQs). The concentration of the environmental pollutants with different biological toxicities can be converted to biological toxicology data based on the TEQ calculation using the monomer TEFs [4,16]. The calculation formula is as follows:

TEQ = ∑ (C_PAHs_ ×TEF) (1)

where C_PAHs_ are the concentrations of individual PAHs.

We used the USEPA standard model [22,23] to assess the Incremental Lifetime Cancer Risk (ILCR) associated with PAH exposure in the topsoil of the study area. People were divided into six groups according to age and gender: adult male and female (18–70 years old), adolescent male and female (11–17 years old), and child male and female (two to 10 years old). The following formulas were used to assess different groups’ ILCR through ingestion, dermal, and inhalation:

(2) $$\mathrm{ILCRsIngestion}=\frac{CS\times (CSF_{Ingestion}\times \sqrt[{3}]{\left(BW/70\right)})\times IR_{\mathrm{soil}}\times EF\times ED}{BW\times AT\times 10^{6}}$$

(3) $$\mathrm{ILCRsDermal}=\frac{CS\times (CSF_{Dermal}\times \sqrt[{3}]{\left(BW/70\right)})\times SA\times AF\times ABS\times EF\times ED}{BW\times AT\times 10^{6}}$$

(4) $$\mathrm{ILCRsInhalation}=\frac{CS\times (CSF_{Inhalation}\times \sqrt[{3}]{\left(BW/70\right)})\times IR_{air}\times EF\times ED}{BW\times AT\times PEF}$$

where CS is the PAH concentration in soil (µg/kg); the other parameters in the formula are defined in Table 1 [2]. The risks for children and adults were calculated separately. The total risk was a sum of the risks associated with each exposure type. We also assessed the exposure risks for construction workers.

In order to calculate the frequency of workers’ daily exposure PAHs, we used the following formula, with reference to Ali’s research [24]:

∑Exposure (ng/kg bw/day) = Cn × IR/body weight
(5)

where IR is the dust ingestion rate, and Cn is the concentrations of PAHs in the CP area soil, respectively.

### 2.7. Statistical Analysis

Excel 2010 (Microsoft Office, Microsoft, Redmond, WA, USA) was used to calculate the descriptive statistics. Isomeric ratios of PAHs were analyzed by Origin 2017 (Originlab, Originlab Corporation, MA, USA). SPSS software (version 16, SPSS, Chicago, IL, USA) was applied to the univariate and multivariate statistical analyses, which were calculated by Principal Component Analysis (PCA).

## 3. Results and Discussion

### 3.1. Concentration and Composition Analysis of PAHs

For the 40 sampling points in the Beijing Coking Plant, the total concentration of 16 priority control PAHs ranged from 314.7 to 1618.3 µg/kg, with an average of 735.3 µg/kg (Table 2). They were all in the same order of magnitude and had an average detection rate of over 90%. All of the samples detected four to six-ring PAHs and other compounds, including Acy, Ace, and DBA. Compared with a previous survey in Xinzhou, the concentration measured in our study was higher than the total amount of target PAHs (202 µg/kg) [25]. The PAH contamination at our study site was much lower than that in Lanzhou (2590 µg/kg) [26] and the Pearl River Delta (1480 µg/kg) [27]. In terms of global comparisons, the PAH concentration in this study was much higher than in Switzerland (225 µg/kg) [28] and Japan (320 µg/kg) [29], but lower than that in India (1906 µg/kg) [30]. The target pollutants studied in the above comparative references were basically 16 priority controlled PAHs. Thus far, there has been no evaluation criteria for PAH pollution in China. According to the total evaluation criteria proposed by Maliszewska-Kordybach [31], 22.5% of the samples were considered heavily polluted (>1000 µg/kg), 30% were considered moderately polluted (600–1000 µg/kg), and 47.5% were considered mildly polluted (200–600 µg/kg).

**Figure 2:**
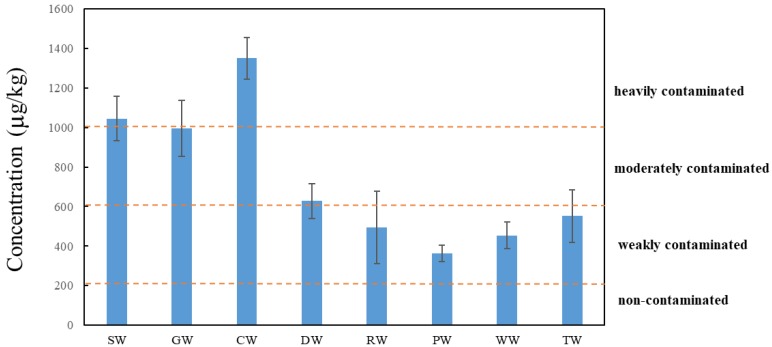
Concentrations of PAHs in surface soil samples (SW, GW, CW, and DW were unrepaired; RW, PW, WW, and TW were repaired). Note: SW: storage workshop; GW: gas workshop; CW: coking workshop; DW: depuration workshop; RW: repair workshop; PW: power workshop; WW: water treatment workshop; TW: tar workshop.

The concentration of PAHs in the surface soil from previous workshops is shown in Figure 2. Although the soils of the RW, PW, WW, and TW workshops had been ectopically repaired between 2010–2013, there still remained certain PAH targets, which may be related to the migration and transformation of pollutants. The total PAH levels in the unrepaired workshops (SW, GW, CW, and DW) were significantly higher than in the repaired workshops. Different levels of pollution were found in all the locations within the study area, with the original CW workshop having the highest concentration (1350.5 µg/kg).

**Figure 3:**
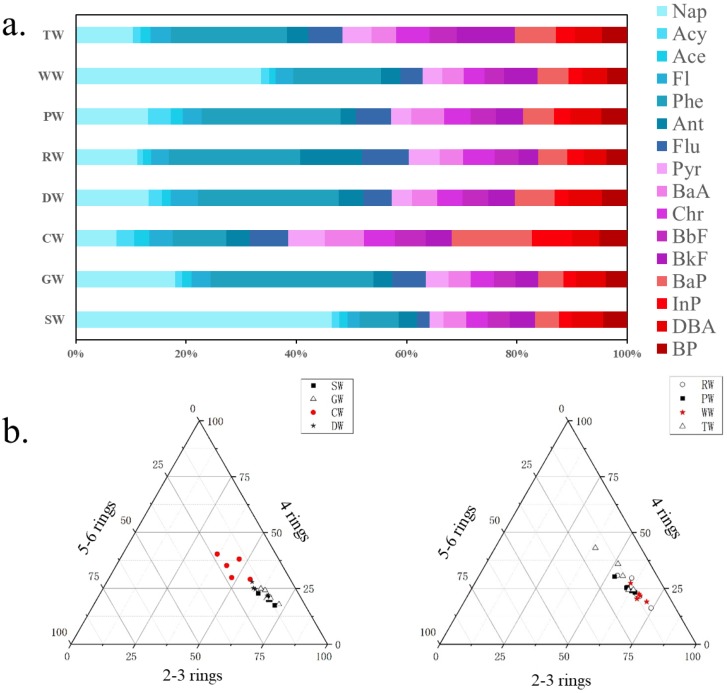
(**a**) The distribution of individual PAHs (the number of rings increases from left to right); (**b**) The relative contributions of PAHs with different numbers of rings.

The 16 PAHs were divided according to the number of rings. These groups included two to three rings (Nap, Acy Ace, Fl, Phe, Ant, and Flu), four rings (Pyr, BaA, Chr, BbF, and BkF) and five to six rings (BaP, DBA, InP, and BP). There were still some variations in the distribution of individual PAHs in the different workshops (Figure 3a). Beside the CW area, PAHs in the other workshops’ soils were dominated by low-ring aromatic hydrocarbons, such as Nap and Phe. The distribution of sampling points in each workshop is shown in Figure 3b. The left schematic represents the RL area, and the right schematic represents the CP area. The distribution of the two soil types is roughly the same and both are higher in the groups with two to three rings and four rings, and lower in the group with five to six rings. The results showed that the PAHs in the surface soil of the study area were mainly those with low rings, which is most likely due to the volatility of the low-ring aromatics.

### 3.2. Source of PAHs in the CP Area

#### 3.2.1. Isomeric Ratios of PAHs

**Figure 4:**
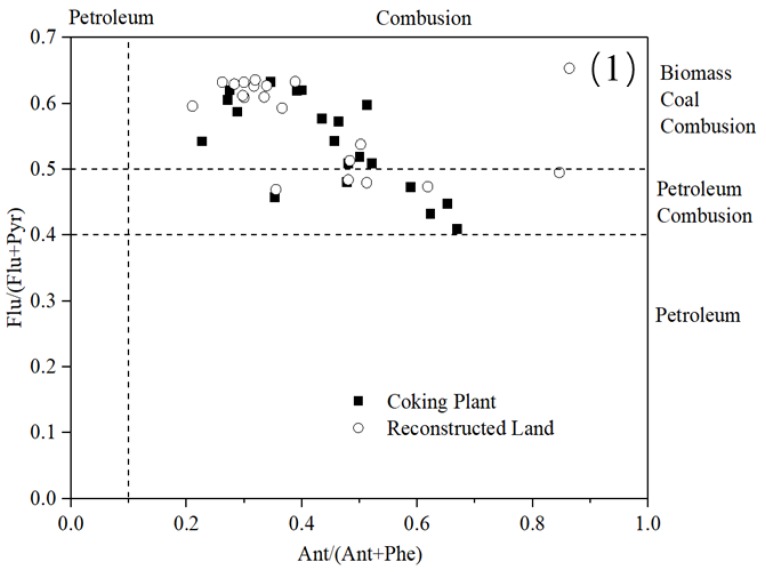

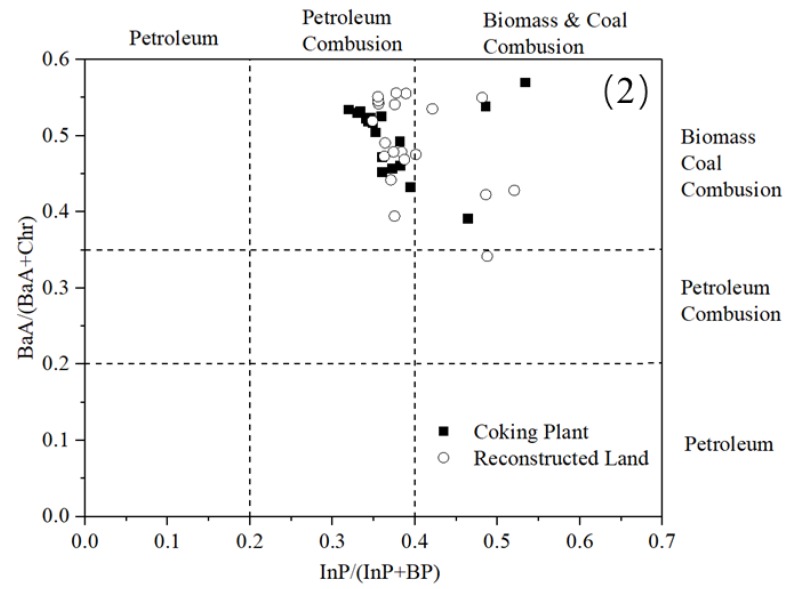
Cross-plots for the individual PAH ratios in the reconstructed land and previous coking plant: (**1**) Ant/(Ant + Phe) versus Flu/(Flu + Pyr) and (**2**) BaA/(BaA + Chr) versus InP/(Inp + BP).

PAHs emitted from different sources exhibit different molecular compositions. The petroleum and combustion are the main anthropogenic sources in the environment. In general, low-molecular weight PAHs are more likely to come from petroleum, while the high-molecular weight PAHs are more likely to come from combustion. There are many PAH source resolution methods, and the isomer ratio method is one of the most commonly used. The 40 sampling points were divided into two parts: the remaining coking plant soil and the reconstructed land. As can be seen in Figure 4, the isomer ratio distribution for the two parts of the topsoil was basically the same, indicating that the target pollutants mostly have strong mobility. Yunker et al. [32] proposed that a ratio of Flu/(Flu + Pyr) between 0.40–0.50 indicated that PAHs are mainly derived from petroleum combustion, while less than 0.40 indicated an oil source, and greater than 0.50 indicated a coal and biomass combustion source. An Ant/(Ant + Phe) ratio of less than 0.1 suggests that the source is petroleum, and a ratio greater than 0.1 suggests that the source is combustion. The PAHs in the surface soil in our study area are primarily from combustion, most of which came from coal and biomass burning, and there was almost no petroleum source. The BaA/(BaA + Chr) ratio was between 0.2–0.35 for petroleum combustion sources, less than 0.2 for petroleum sources, and greater than 0.35 for combustion sources. The InP/(Inp + BP) ratio was between 0.2–0.4 for petroleum combustion sources, less than 0.2 for petroleum sources, and greater than 0.4 for combustion sources. Figure 4(2) again demonstrates that the soil PAHs at the sampling points are mainly derived from the combustion of coal and biomass. Petroleum fuels and products are not an important source of PAHs in the surface soil of the coking plant.

#### 3.2.2. Principal Component Analysis

**Figure 5:**
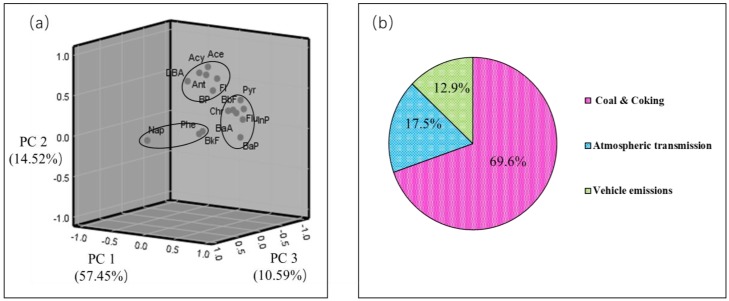
(**a**) Factor loadings and (**b**) the contributions of the three major sources of PAHs in surface soil samples.

Principal component analysis (PCA) is a multivariate statistical method that uses multiple variables to linearly transform and select fewer significant variables. In this study, 16 factors were selected, including Nap, Acy, Ace, Fl, Phe, Ant, Flu, Pyr, BaA, Chr, BbF, BkF, BaF, InP, DBA, and BP. Through KMO (Kaiser–Meyer–Olkin) and Sig (Significance) tests, we concluded that the KMO value reached 0.85 and the SIG was 0, which indicates that the data were taken from the normal distribution, the correlation between variables was recognized, and the data were suitable for factor analysis. The PCA results showed that 82.56% of the total difference was calculated from the first three main factors. PC1 accounted for 57.45% of the total variance, and was dominated by Flu, Byr, Chr, BbF, BaA, and InP (Figure 5a). In this group, the load for Flu and Pyr was significantly higher than that of other compounds, which are indicators of coal combustion [33]. PC2 accounts for 14.52% of the total variance, and was characterized by Nap, which typically represents atmospheric transmission [34], and indicated that there were external pollutant sources. In PC3, Acy, Ace, and Ant were mainly from the low-temperature combustion of petroleum fuel, which is an indicator of traffic emissions [35,36]. PCA showed that the soil PAHs were mainly derived from coal combustion, while traffic pollution and external mixed sources accounted for an additional proportion. Figure 5b is a conclusion based on the isomeric ratios and PCA, indicating that the source of PAHs in the region were mainly traffic pollution and atmospheric transmission.

### 3.3. Health Risk Assessment

#### 3.3.1. Toxicity Assessment of Pollutants

The combination of different pollutants can enhance or diminish toxicity. Mixing multiple pollutants can produce lower or higher toxicity than that of single pollutant. Therefore, it is necessary to consider the toxicity of various pollutants comprehensively to evaluate the risk in our study area. The study area was divided into two parts for comparison: the soil after restoration of the reconstructed land, and the unrepaired soil of the original coking plant. The total TEQ for the 16 PAHs varied from 39.4 to 559.5 µg/kg, and averaged 108.8 µg/kg, which was slightly less than the value of Lanzhou (138 µg/kg) [26]. The total TEQ for 10 PAHs in the Dutch soil standard was between 2.54–83.82 µg/kg, with an average of 27.33 µg/kg. All of the sampling points in our study area exceeded the Dutch soil standard target reference value (33.0 µg/kg).

As can be seen from Table 3, the average TEQ for the 16 PAHs in the RL area was 60.1 µg/kg, and was much less than that in the CP area (157.6 µg/kg). The seven highly carcinogenic PAHs accounted for 98.3% and 99.1% of the PAHs in the two regions, indicating that the seven carcinogenic PAHs in the two regions were the main contributors to total TEQ. Both showed that BaP and DBA contributed significantly to the total TEQ value, and accounted for 46.7% and 36.7% of the RL area and 52.5% and 34.2% of the CP area, respectively. Therefore, the two PAHs in the study area should be taken seriously.

#### 3.3.2. Biological Genotoxicity Analysis

The genotoxicity test proved that the luminescence intensity of the bioreporter and the content of MMC and BaP in contaminated soil satisfied the dose–response relationship. The higher induction ratio indicates greater genotoxicity. This can be used to evaluate the genotoxicity of soil contaminated with PAHs.

**Figure 6:**
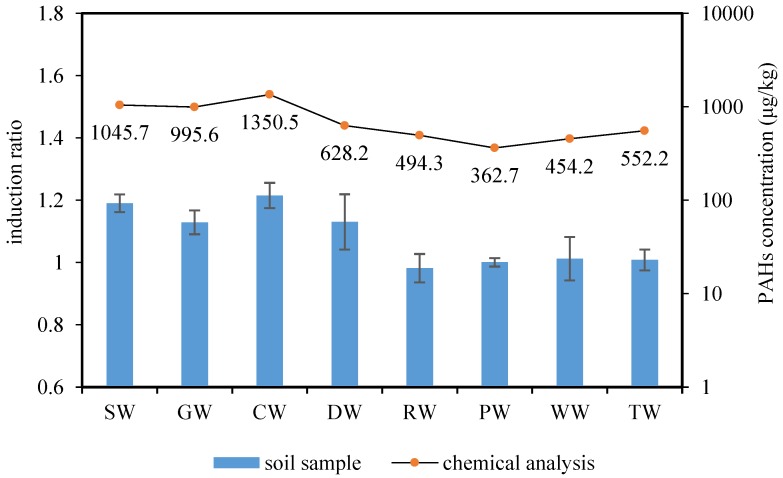
Comparison of genotoxicity (column) and chemical analysis (dots) of soil samples. Error bars indicate standard deviations of the replicates.

The results of the ADP1-recA toxicity analysis for soil samples from different workshops are shown in Figure 6. The induction ratios of the RW, PW, WW, and TW workshops were less than or equal to one, indicating no genotoxicity. The induction ratios of the SW, GW, CW, and DW workshops were significantly greater than one, indicating a certain degree of genotoxicity. These results should be taken seriously, and the CW region contained the greatest genotoxicity. These were consistent with the results from the chemical analysis. Some specific samples were different from chemical results, such as the genotoxicity of GW and DW, where large differences in chemical results were approximately equal. This further proved that the cooperation and antagonism between different pollutants may cause differences in bioavailability.

#### 3.3.3. Lifetime Cancer Risk Assessment of the Reconstructed Regional Population

In this study, ILCR of PAHs in the RL area were used to assess the health risk caused by restored soil for the residential populations. At the same time, the CP area will also be turned into residential land. We studied the daily exposure of construction workers in the soil environment to assess the health impact of PAHs during project construction.

ILCRs of 10^−6^ or less are considered to have a negligible risk, while values ranging from 10^−6^ to 10^−4^ denote potential health risks. ILCRs greater than 10^−4^ indicate higher risks. The carcinogenic risk values through direct ingestion, dermal contact, and inhalation ranged from 1.03 × 10^−6^ to 8.86 × 10^−6^, 1.06 × 10^−6^ to 1.52 × 10^−4^, and 8.20 × 10^−8^ to 1.21 × 10^−6^, respectively. Table 4 demonstrates that breathing, ingestion, and dermal contact of the reconstructed area in the coking plant had potential carcinogenic risks for different age groups, and the ILCRs were calculated based on average concentration. For different age groups, the ILCRs for adults were higher than those for children and adolescents, while, within the same age group, those for females were higher than those for males. In general, different age groups had different potential cancer risks.

**Figure 7:**
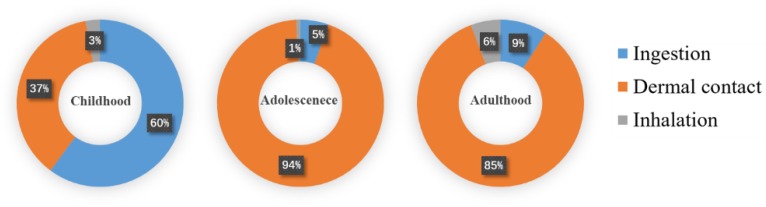
Cumulative probability of ILCRs in different exposure pathways for childhood, adolescence, and adulthood.

As presented in the Figure 7, different exposure pathways had different effects on different age groups. For children, the risk of direct ingestion was higher than that of dermal contact and inhalation. For adolescents and adults, the risks decreased as follows: dermal contact > direct ingestion > inhalation. The health risks between different genders were not distinct. In reality, the ILCR for the worst-case scenario remained in the same order of magnitude as the average exposures. In the reconstructed area, the integrated lifetime cancer risks associated with exposure to soils with average PAH concentrations for different populations are acceptable.

#### 3.3.4. Calculation of Daily Ingestion of Construction Workers

Since the CP area and the RL area are now well-isolated—the CP area has been fenced as a construction site, and the coking plant has been relocated for many years—this study separately discussed its risk for residents and workers. Construction workers ingest surface soil dust through skin, the mouth, and breath, which is considered to be one of the main ways people are exposed to multiple pollutants per day [33]. The contribution of Nap (0.07–0.81 ng/kg·bw/day) and Phe (0.03–0.52 ng/kg·bw/day) was significant via dust intake, which may be related to the volatility of low-ring aromatics. BaP content (0.04–0.72 ng/kg·bw/day) was higher for carcinogenic PAHs in the sample, which may pose long-term health risks. The total intake of 16 PAHs for construction workers was between 0.74–2.31 ng/kg·bw/day (shown in Table 5), which was less than the exposure in the vehicle factory according to Ali et al. [37]. At the same time, it can be seen that Nap and Phe still occupied the main part of the contact intake of 16 PAHs, which should be related to their high volatility; meanwhile, in high-ring aromatics, BaP was still the main force and the main contributor to cancer risk. The lower ingestion may be related to the air circulation associated with outdoor work. Since China’s big cities such as Beijing are now using clean production in the construction process, the chances of workers being exposed to pollutants have been greatly reduced. However, due to the small sample size and the limitations of the study area, the range of estimated exposure only pertained to adult construction workers. This study highlights the effects of neglecting reconstructed industrial sites and the importance of monitoring workers’ health risks when exposed to environmental pollutants.

## 4. Conclusions

With the expansion of large cities and the relocation of factories, the problem of soil pollution on land previously occupied by factories has attracted more attention. This study analyzed the pollution level and source of PAHs in the surface soil of the Beijing Coking Plant remodeling area. This study was a preliminary discussion on the pollution of PAHs in the reconstruction of old industrial sites, and has a certain application value for the risk assessment of construction land. After all, research on a single industrial site is not very universal, but it can provide some methods and ideas for future research. The total concentration of 16 PAHs ranged from 314.7 to 1618.3 µg/kg, with an average of 735.3 µg/kg. Low-ring aromatics (two to three rings) dominated the PAH profile, and the diagnostic ratio and PCA indicated that coal combustion might be the main source of residual PAHs. Our results indicate that PAHs were still mainly derived from the coking plant. The genotoxicity of the study area was within the acceptable range. The overall lifetime cancer risk for different age groups was between 4.35 × 10^−6^ and 5.64 × 10^−5^; there was a potential cancer risk for the people in the RL area. The total intake of 16 PAHs was between 0.74–2.31 ng/kg bw/day, of which Nap and Phe were the main contributors. This research explored the concentration, source, and risk level for PAHs in the surface soil of the remodeling coking plant. To confront the serious environmental issues, this study could provide new insights for the soil risk management of remodeled industrial sites contaminated with PAHs or other organic pollutants.

## Figures and Tables

**Table 1:** Parameters used in the incremental lifetime cancer risk assessment.

Parameter	Unit	Child		Adolescent		Adult	
		Male	Female	Male	Female	Male	Female
Body weight (BW)	kg	17.2	16.5	47.1	44.8	60.2	53.1
Exposure frequency (EF)	d·year^−1^	350	350	350	350	350	350
Exposure duration (ED)	year	6	6	14	14	30	30
Inhalation rate (IRair)	m^3^·d^−1^	10.9	10.9	17.7	17.7	17.5	17.5
Soil intake rate (IRsoil)	mg d^−1^	200	200	100	100	100	100
Dermal surface exposure (SA)	cm^2^·d^−1^	1800	1800	5000	5000	5000	5000
Averaging life span (AT)	year	25,550	25,550	25,550	25,550	25,550	25,550
Soil dust produce factor (PEF)	m^3^·kg^−1^	6.2 × 10^9^	6.2 × 10^9^	6.2 × 10^9^	6.2 × 10^9^	6.2 × 10^9^	6.2 × 10^9^
Carcinogenic slope factor (CSF) Ingestion	(mg·kg^−1^·d^−1^)^−1^	7.3	7.3	7.3	7.3	7.3	7.3
Carcinogenic slope factor (CSF) Dermal	(mg·kg^−1^·d^−1^)^−1^	25	25	25	25	25	25
Carcinogenic slope factor (CSF) Inhalation	(mg·kg^−1^·d^−1^)^−1^	3.85	3.85	3.85	3.85	3.85	3.85

**Table 2:** Detection of 16 polycyclic aromatic hydrocarbons (PAHs) in coking plant soils (dry weight, *n* = 40, µg/kg).

PAHs	Abbreviation	Max	Min	Mean	SD	Detectable (%)
Naphthalene	Nap	567.5	8.6	144.8	132.7	97.3
Acenapthylene	Acy	89.1	4.2	14.9	55.4	100
Acenapthene	Ace	71.7	3.4	14.1	62.6	100
Fluorene	Fl	90.5	7.6	26.1	59.2	96.5
Phenanthrene	Phe	365.9	22.4	132.3	214.8	96.5
Anthracene	Ant	128.1	9.4	31.9	73.1	96.5
Fluoranthene	Flu	116.9	10.7	41.1	85.6	100
Pyrene	Pyr	103.4	9.2	33.3	72.5	100
* Benzop[a]anthracene	BaA	178.9	13.7	36.5	134.2	100
* Chrysene	Chr	113.8	12.7	35.5	69.3	100
* Ben[b]fluoranthene	BbF	91.7	13.3	33.7	73.5	93.6
* Ben[k]fluoranthene	BkF	174.9	15.4	37.9	96.4	100
* Benzo[a]pyrene	BaP	502.2	13.9	55.6	239.5	93.6
* Indeno[1,2,3-cd]pyrene	InP	213.1	7.8	27.1	164.6	100
* Dibenzo[a,h]anthracene	DBA	109.6	18.7	38.3	45.8	100
Benzo[g,h,i]perylene	BP	89.6	14.5	32.5	55.8	100
∑16PAHs	-	3006.9	185.5	735.6	893.7	-
∑Carcinogenic 7PAHs	-	1384.2	95.5	264.6	532.6	-

* Carcinogenic PAH.

**Table 3:** Toxic equivalent concentration (BaPeq) (µg/kg) of PAHs in soils from the study areas.

PAHs	Abbreviation	TEFs	BaPeq	
			RL	CP
Naphthalene	Nap	0.001	0.07 ± 0.04	0.21 ± 0.07
Acenapthylene	Acy	0.001	0.03 ± 0.01	0.06 ± 0.01
Acenapthene	Ace	0.001	0.02 ± 0.01	0.03 ± 0.01
Fluorene	Fl	0.001	0.02 ± 0.01	0.04 ± 0.01
Phenanthrene	Phe	0.001	0.1 ± 0.04	0.17 ± 0.04
Anthracene	Ant	0.01	0.5 ± 0.25	0.38 ± 0.03
Fluoranthene	Flu	0.001	0.03 ± 0.01	0.05 ± 0.01
Pyrene	Pyr	0.001	0.02 ± 0.01	0.05 ± 0.01
* Benzop[a]anthracene	BaA	0.1	2.13 ± 0.63	5.17 ± 0.18
* Chrysene	Chr	0.01	0.24 ± 0.09	0.47 ± 0.06
* Ben[b]fluoranthene	BbF	0.1	2.05 ± 1.12	4.68 ± 0.28
* Ben[k]fluoranthene	BkF	0.1	3.02 ± 0.21	4.57 ± 0.16
* Benzo[a]pyrene	BaP	1	28.22 ± 5.97	83.04 ± 1.86
* Indeno[1,2,3-cd]pyrene	InP	0.1	6.38 ± 0.77	4.03 ± 0.13
* Dibenzo[a,h]anthracene	DBA	1	22.21 ± 4.34	54.29 ± 2.71
Benzo[g,h,i]perylene	BP	0.01	0.19 ± 0.05	0.47 ± 0.03
Σ16PAHs	-	-	60.1 ± 19.9	157.6 ± 33.1

* Carcinogenic PAH.

**Table 4:** Incremental Lifetime Cancer Risks (ILCRs) of people for different exposure pathways.

Exposure pathways	Childhood		Adolescence		Adulthood	
	Male	Female	Male	Female	Male	Female
Ingestion	2.62 × 10^−6^	2.69 × 10^−6^	1.56 × 10^−6^	1.61 × 10^−6^	2.84 × 10^−6^	3.08 × 10^−6^
Dermal contact	1.62 × 10^−6^	1.67 × 10^−6^	2.67 × 10^−5^	2.76 × 10^−5^	4.86 × 10^−5^	5.29 × 10^−5^
Inhalation	1.21 × 10^−7^	1.25 × 10^−7^	2.35 × 10^−7^	2.43 × 10^−7^	4.23 × 10^−7^	4.59 × 10^−7^
Total ILCRs	4.35 × 10^−6^	4.48 × 10^−6^	2.85 × 10^−5^	2.95 × 10^−5^	5.18 × 10^−5^	5.64 × 10^−5^

**Table 5:** Assessment of daily exposure via dust ingestion for construction workers.

Sample ID	Nap	Acy	Ace	Fl	Phe	Ant	Flu	Pyr	BaA	Chr	BbF	BkF	BaP	InP	DBA	BP	Σ16PAHs
C 1-1	0.61	0.02	0.02	0.04	0.12	0.04	0.03	0.03	0.06	0.05	0.05	0.06	0.06	0.03	0.08	0.06	1.38
C 1-2	0.65	0.02	0.02	0.05	0.16	0.05	0.06	0.05	0.06	0.07	0.06	0.07	0.06	0.04	0.08	0.06	1.57
C 1-3	0.81	0.02	0.02	0.02	0.05	0.05	0.03	0.04	0.06	0.06	0.07	0.07	0.07	0.04	0.09	0.07	1.55
C 1-4	0.59	0.02	0.01	0.02	0.03	0.04	0.02	0.03	0.06	0.05	0.06	0.07	0.07	0.03	0.09	0.07	1.28
C 1-5	0.79	0.02	0.05	0.03	0.16	0.05	0.03	0.03	0.06	0.05	0.06	0.07	0.07	0.03	0.09	0.07	1.68
C 2-1	0.29	0.01	0.01	0.04	0.36	0.05	0.05	0.05	0.05	0.05	0.05	0.06	0.06	0.03	0.08	0.06	1.28
C 2-2	0.19	0.01	0.02	0.05	0.52	0.04	0.10	0.06	0.05	0.05	0.05	0.06	0.06	0.03	0.08	0.05	1.43
C 2-3	0.47	0.03	0.05	0.07	0.44	0.05	0.09	0.05	0.06	0.07	0.05	0.06	0.07	0.03	0.08	0.06	1.72
C 2-4	0.16	0.01	0.02	0.03	0.34	0.06	0.08	0.06	0.06	0.06	0.05	0.05	0.06	0.03	0.07	0.05	1.20
C 2-5	0.18	0.01	0.04	0.06	0.43	0.05	0.12	0.08	0.06	0.08	0.07	0.06	0.09	0.04	0.07	0.06	1.49
C 3-1	0.19	0.13	0.10	0.13	0.12	0.12	0.13	0.12	0.14	0.11	0.10	0.10	0.11	0.15	0.16	0.13	2.03
C 3-2	0.12	0.09	0.07	0.10	0.10	0.13	0.14	0.15	0.13	0.11	0.12	0.09	0.13	0.11	0.14	0.12	1.84
C 3-3	0.10	0.03	0.04	0.05	0.30	0.05	0.12	0.09	0.08	0.02	0.12	0.06	0.72	0.08	0.05	0.07	1.96
C 3-4	0.17	0.03	0.03	0.07	0.23	0.05	0.17	0.14	0.26	0.16	0.13	0.13	0.28	0.30	0.06	0.10	2.31
C 3-5	0.13	0.04	0.03	0.06	0.19	0.07	0.12	0.14	0.09	0.13	0.08	0.07	0.16	0.06	0.07	0.07	1.51
C 4-1	0.17	0.03	0.01	0.05	0.12	0.04	0.04	0.03	0.05	0.05	0.06	0.04	0.15	0.03	0.05	0.04	0.96
C 4-2	0.15	0.02	0.02	0.06	0.30	0.05	0.06	0.04	0.04	0.05	0.04	0.04	0.04	0.02	0.05	0.04	1.03
C 4-3	0.09	0.02	0.01	0.02	0.21	0.03	0.04	0.02	0.04	0.04	0.04	0.04	0.04	0.02	0.05	0.04	0.74
C 4-4	0.07	0.02	0.02	0.06	0.32	0.06	0.06	0.04	0.04	0.04	0.04	0.04	0.04	0.02	0.05	0.04	0.96
C 4-5	0.11	0.02	0.01	0.02	0.20	0.03	0.04	0.03	0.04	0.04	0.04	0.04	0.04	0.02	0.05	0.04	0.79
MAX	0.81	0.13	0.10	0.13	0.52	0.13	0.17	0.15	0.26	0.16	0.13	0.13	0.72	0.30	0.16	0.13	2.31
MIN	0.07	0.01	0.01	0.02	0.03	0.03	0.02	0.02	0.04	0.02	0.04	0.04	0.04	0.02	0.05	0.04	0.74

Note: Nap: naphthalene; Acy: acenaphthylene; Ace: acenaphthene; Fl: fluorene; Phe: phenanthrene; Ant: anthracene; Flu: fluoranthene; Pyr: pyrene; BaA: benz[a]anthracene; Chr: chrysene; BbF: benzo[b]fluoranthene; BkF: benzo[k]fluoranthene; BaP: benzo[a]pyrene; InP: indeno[1,2,3-cd]pyrene; DBA: dibenz[a,h]anthracene; BP: benzo[g,h,i]perylene.

## Supplementary Materials

The following are available online at https://www.mdpi.com/1660-4601/16/4/670/s1, Table S1: Selective ions, Linear equations and Peak time of 16 PAHs.
