# Supplementary material for: Contamination, Sources, and Health Risks Associated with Soil PAHs in Rebuilt Land from a Coking Plant, Beijing, China
Source: Int J Environ Res Public Health. 2019 Feb 25;16(4):670. doi: 10.3390/ijerph16040670 (PMC6407006; doi:10.3390/ijerph16040670)
Supplement: Supplementary file 1 [file ijerph-16-00670-s001.pdf]

International Journal of Environmental Research and Public Health

Supplementary material

Contamination, sources and health risks associated with soil PAHs in rebuilt land from a Coking Plant, Beijing, China

Wei Cao [1], Liqin Yin [1], Dan Zhang [2], Yingying Wang [1], Jing Yuan [1], Yi Zhu [1,*] and Junfeng Dou [1,*]

[1] College of Water Sciences, Beijing Normal University, Beijing 100875, China; caowei@mail.bnu.edu.cn (W.C.); 18435154253@163.com (L.Y.); wangyingyingfei@126.com (Y.W.); xyvy-8945@163.com (J.Y.)

[2] Beijing Municipal Research Institute of Environmental Protection, National Engineering Research Center of Urban Environmental Pollution Control, Beijing Key Laboratory for risk modeling and remediation of contaminated sites, Beijing 100037, China; zhangdan615@126.com (D.Z.)

* Correspondence: doujf@bnu.edu.cn; Tel.: +86-10-5880-2739 (J.F.D.); zhuyi@bnu.edu.cn; Tel.: +86-10-5880-4198 (Y.Z.)

International Journal of
Environmental Research and Public Health

MDPI

Table S1. Selective ions, Linear equations and Peak time of 16 PAHs.

Compound	Selective Ions (m/z)	Standard Curve Equation	Linear Coefficient	Peak Time (min)
Naphthalene (Nap)	102,128	y = 0.0075x + 0.86	0.9992	6.8–7.2
Acenapthylene (Acy)	76,126,152	y = 0.0068x - 4.12	0.9972	12.4–12.8
Acenapthene (Ace)	76,126,153	y = 0.0046x - 3.66	0.9964	13.2–13.6
Fluorene (Fl)	82,139,166	y = 0.0041x - 3.72	0.9982	15.8–16.2
Phenaathrene (Phe)	89,152,178	y = 0.0084x - 0.97	0.9987	20.6–21.0
Anthracene (Ant)	89,152,178	y = 0.0083x +1.39	0.9921	21.0–21.2
Fluoranthene (Flu)	101,174,202	y = 0.0064x - 0.23	0.9906	27.2–27.8
Pyrene (Pyr)	101,174,202	y = 0.0088x - 1.84	0.9924	28.4–29.0
Benzop[a]anthracene (BaA)	114,200,228	y = 0.0032x - 2.48	0.9997	35.4–35.9
Chrysene (Chr)	113,200,228	y = 0.0072x - 2.75	0.9966	35.9–36.2
Ben[b]fluoranthene (BbF)	126,224,252	y = 0.0056x - 0.25	0.9917	41.4–41.7
Ben[k]fluoranthene (BkF)	126,224,252	y = 0.0067x - 0.34	0.9905	41.7–42.0
Benzo[a]pyrene (BaP)	126,224,252	y = 0.0059x - 0.25	0.9993	42.8–43.4
Indeno[1,2,3-cd]pyrene (InP)	138,276	y = 0.0045x - 0.21	0.9967	48.0–48.3
Dibenzo[a,h]anthracene (DBA)	139,278	y = 0.0025x - 0.06	0.9951	48.3–48.8
Benzo[g,h,i]perylene (BP)	138,276	y = 0.0042x - 0.19	0.9946	49.0–49.6
